# Synovial fluid and radiographic evaluation of joints from dogs with visceral leishmaniasis

**DOI:** 10.1186/s13071-022-05444-y

**Published:** 2022-09-08

**Authors:** Alexandre R. S. Silva, Ana A. D. Gomes, Monally C. C. Aquino, Breno F. M. Almeida, Valéria M. F. Lima, Paulo C. Ciarlini, Luciana D. R. Pinoti, Mary Marcondes, Rafael F. C. Vieira

**Affiliations:** 1 Center of Agrarian Sciences, Universidade Federal do Vale do São Francisco (UNIVASF), Petrolina, Pernambuco Brazil; 2 Universidade Estácio de Sá (UNESA), Rio de Janeiro, Brazil; 3 University Center of the Integrated Faculties of Ourinhos (Unifio), Ourinhos, São Paulo, Brazil; 4 School of Veterinary Medicine, São Paulo State University (UNESP), Araçatuba, São Paulo, Brazil; 5 Vector-Borne Diseases Laboratory, Departament of Veterinary Medicine, Universidade Federal do Paraná (UFPR), Curitiba, Paraná Brazil; 6 Global One Health Initiative (GOHi), The Ohio State University, Columbus, OH USA

**Keywords:** Polyarthritis, *Leishmania infantum*, *Ehrlichia canis*

## Abstract

**Background:**

Polyarthritis has been associated with canine visceral leishmaniasis (CanVL), and co-infection with *Ehrlichia canis* is common and may alter clinical manifestations.

**Methods:**

A total of 89 dogs presenting CanVL were subdivided into two groups: (1) G1, consisting of 46 dogs seronegative to *Ehrlichia* spp., and (ii) G2, consisting of 43 dogs seropositive to *Ehrlichia* spp. Eight joints (carpal, tarsal, stifles and elbows) from each dog were evaluated by radiography and synovial fluid (SF) cytologic analysis.

**Results:**

Overall, 74 of the 89 (83.1%) dogs presented joint abnormalities suggestive of osteoarthritis by radiography (G1: 40/46 [86.9%]; G2: 34/43 [79.0%]), with no statistically significant between-group difference. All dogs with abnormal joint X-ray images presented radiographic lesions bilaterally, independent of the characteristics of the lesion. Soft tissue swelling around the joint and joint space narrowing were more commonly observed in G1 than in G2 dogs. There was no significant between-group difference in terms of other radiographic abnormalities suggestive of osteoarthritis (evident trabecular pattern, subchondral bone sclerosis, osteolysis, osteolytic–proliferative lesions or bone proliferation). SF from 174/315 (55.2%) and 152/307 (49.5%) joints from G1 and G2 dogs, respectively, presented an inflammatory infiltrate, but there was no significant association between the presence of inflammatory infiltrate and group. There was also no statistical difference between groups in either of the evaluated joints in terms of the percentage of neutrophils or mononuclear cells. *Leishmania* spp. amastigotes were found in 69/315 (21.9%) joints from G1 dogs and in 100/307 (32.5%) joints from G2 dogs (Fisherʼs exact test, *P* = 0.002, odds ratio = 0.5, 95% confidence interval = 0.4–0.8). The neutrophilic infiltrate was significantly higher in joints with amastigote forms in both G1 (Mann–Whitney U-test, *U*_(18)_ = 817, *Z* = -3.76, *P* = 0.0001) and G2 dogs (Mann–Whitney U-test, *U*_(18)_ = 6543, *Z* = − 5.06, *P* < 0.0001).

**Conclusions:**

A high prevalence of arthritis in dogs with CanVL was found, and all dogs presented involvement in multiple joints. Although no difference was observed between groups in terms of the number of dogs with polyarthritis and the presence of an inflammatory infiltrate in SF, *Leishmania* spp. amastigotes were found more frequently in joints from G2 dogs. Further studies evaluating SF in dogs co-infected with *L. infantum* and *E. canis* should be performed to evaluate this finding.

**Graphical Abstract:**

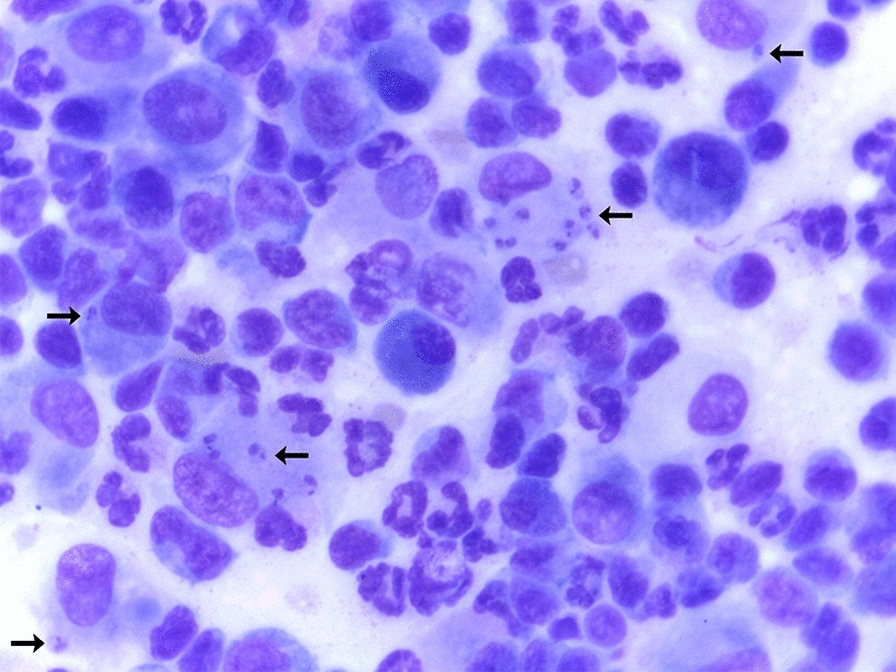

## Background

Canine visceral leishmaniasis (CanVL) is a chronic and progressive disease caused by *Leishmania infantum*. Its presentation is variable and can include such clinical signs as fever, progressive weight loss, peripheral lymphadenopathy, splenomegaly, dermatological lesions, onychogryphosis, epistaxis, vomiting and diarrhea, in addition to renal, ophthalmic, neurological and musculoskeletal manifestations [1, 2]. In endemic areas for CanVL, co-infections of *L. infantum* and other vector-borne pathogens, such as *Ehrlichia canis*, are common and may alter clinical manifestations, complicating diagnosis, treatment and prognosis [3].

Arthritis and polyarthritis have been associated with CanVL and may be caused by two mechanisms, the direct presence of *L. infantum* amastigotes and/or a type III hypersensitivity reaction with deposition of immune complex within the joint [4, 5]. These two mechanisms are probably interrelated and may coexist in the same joint [6]. *Leishmania infantum* can cause either non-erosive [4, 7–9] or erosive [4, 8, 10–12] arthritis. While the presence of bone and joint lesions due to CanVL in plain radiographs has been reported, computed tomography findings have suggested that joint lesions may be common in CanVL but not so commonly described because of the subclinical characteristic observed in most cases [13].

*Ehrlichia canis*-related polyarthritis in dogs with detection of *Ehrlichia morulae* in the synovial fluid (SF) has been reported in dogs, but only rarely [14, 15], and there is no conclusive evidence supporting the occurrence of arthritis [16]. In addition, SF evaluation revealed no cytological evidence of arthritis in dogs with experimental acute canine monocytic ehrlichiosis (CME) [17]. Accordingly, the aim of this study was to evaluate the joint involvement pattern, by radiography and SF cytologic examination, of dogs presenting CanVL with and without the presence of anti-*Ehrlichia* spp. antibodies.

## Methods

### Animal selection and CanVL diagnosis

A total of 89 adult dogs naturally affected and presenting clinical signs of CanVL that had been referred to the Center for Zoonosis Control of Araçatuba (21°12′32″ S and 50°25′58″ W), a municipality with high endemicity for CanVL in São Paulo State, southern Brazil, were evaluated. CanVL diagnosis was based on the direct observation of *Leishmania* amastigote forms in smears from lymph node aspirates obtained by fine-needle aspiration and stained with a quick Romanovsky-type stain (Panótico Rápido®; Laborclin, Pinhais, Brazil), and on serology by using indirect enzyme-linked immunosorbent assay (ELISA) kit (Bio-Manguinhos/Fiocruz, Rio de Janeiro, Brazil). The ELISA is based on microtiter plates previously sensitized with soluble antigens of *Leishmania major* (e.g. MHOM/BR/76/JOF) and is used by the Brazilian Ministry of Health for the diagnosis of CanVL. The inclusion criterion for the study was positive test results for CanVL in both diagnostic tests. In compliance with the Brazilian federal law at the time of the study, dogs were euthanized after *L. infantum* diagnosis. All dogs underwent radiographic examination and arthrocentesis of eight different joints before euthanasia.

All dogs were tested for the presence of anti-*Ehrlichia* spp., anti-*Anaplasma* spp. and anti-*Borrelia burgdorferi* antibodies by a commercial ELISA rapid test (SNAP® 3Dx®; IDEXX Laboratories Inc., Westbrook, ME, USA), according to the manufacturer’s instructions. All serum samples tested negative for anti-*Anaplasma* spp. and anti-*B. burgdorferi* antibodies. According to the presence or absence of anti-*Ehrlichia* spp. antibodies, dogs were subdivided into two groups: (i) Group 1 (G1), which included 46 dogs with CanVL and seronegative to *Ehrlichia* spp.; and (ii) Group 2 (G2), which included 43 dogs with CanVL and seropositive to *Ehrlichia* spp.

### Imaging studies

In both the right and left limbs, craniocaudal and mediolateral projections of the elbow and stifle joints and dorsopalmar/dorsoplantar and mediolateral projections of the carpal and tarsal joints were performed with an X-ray system (SHF 730; CRX, São Paulo, Brazil), with total imaging of eight joints per dog. The parameters were a focus-film distance of 10 cm; 10 mAs and variable kilovoltage peak (kVp; range: 50–70) according to the measured thickness of each joint region. Films were automatically processed using an MX2 processor (Macrotec, São Paulo, Brazil). Each joint was examined for the presence of lesions, type of lesions and involvement of adjacent soft tissues. Two experienced veterinary radiologists evaluated all images.

### SF examination

Aseptic arthrocentesis of elbows, carpals, stifles and tarsal joints were performed using a fine needle (25 × 0.7 mm) and a 5-ml sterile syringe, and the maximum amount of SF was collected. SF was obtained by arthrocentesis of at least four joints of each dog. SF samples from the remaining joints were discarded due to insufficient volume or the presence of blood in the sample. The slides were prepared immediately after SF collection by cytocentrifugation at 380* g* for 5 min. Direct cytologic examination was performed on smears stained by a quick Romanovsky-type stain (Panótico Rápido®; Laborclin). Ten fields were examined in each smear, and the cell count was recorded. The percentage of nucleated cells (neutrophils, lymphocytes, monocytes and macrophages) was calculated, and the presence of *Leishmania* spp. amastigotes was determined under a light microscope at ×1000 magnification. The SF was considered to be normal if no more than 3 cells/field at ×1000 magnification were noted [18].

### Statistical analysis

Descriptive data were tabulated in an electronic spreadsheet (Excel®; Microsoft Corp., Redmond, WA, USA), with absolute and relative (%) frequencies of radiographic and SF findings determined. Comparisons between groups were performed after analyzing the variables for normality (Shapiro–Wilk test) using the Mann–Whitney test. Associations between categorical variables were determined by Fisher's exact test with the determination of the odds ratio (OR) and 95% confidence interval (CI). Analyses were performed using commercially available statistical software (InStat version 6.00; GraphPad Software, San Diego, CA, USA) and considered significant when *P* < 0.05.

## Results

### Animals and groups

The dogs included in this study had been referred to the Municipal Zoonoses Control Center due to a diagnosis of CanVL at a time when euthanasia of these animals was recommended in Brazil; consequently, it was not possible to obtain their clinical history. However, all dogs presented multiple clinical signs of CanVL at the time of sampling, such as weight loss, skin lesions, onychogryphosis, epistaxis, lymphadenopathy and diarrhea.

Of the 46 G1 dogs, 21 (45.6%) were male and 25 (54.3%) were female. There were 32 (69.5%) mongrels and 14 (30.4%) pure-breed dogs (Doberman Pinscher, *n* = 3; Poodle, *n* = 2; Cocker Spaniel, *n* = 2; Boxer, *n* = 2; Rottweiler, *n* = 2; Bull terrier, *n* = 1; Yorkshire terrier, *n* = 1; Labrador retriever, *n* = 1). The age of G1 dogs ranged from 1 to 12 (median: 3) years, with 32 dogs (69.5%) aged between 1 and 3 years, 12 (26.0%) aged between 4 and 7 years and two (4.3%) aged > 8 years.

Of the 43 G2 dogs, 28 (65.1%) were male and 15 (34.8%) were female. There were 28 (65.1%) mongrels and 15 (34.8%) pure-breed dogs (Poodle, *n* = 5; Dachshund, *n* = 5; Brazilian terrier, *n* = 3; Cocker Spaniel, *n* = 1; Maltese, *n* = 1). The age of G2 dogs ranged from 1 to 12 (median: 3) years, with 24 (55.8%) dogs aged between 1 and 3 years, 17 (39.5%) dogs aged between 4 and 7 years and two (4.6%) dogs aged > 8 years.

### Radiographic evaluation

Overall, of the 89 dogs evaluated in the study, 74 (83.1%, 95% CI: 74.0–89.5) presented joint abnormalities on radiographic images suggestive of osteoarthritis: 40/46 (86.9%; 95% CI = 74.3–93.8) in G1 and 34/43 (79.0%; 95% CI = 64.7–88.5) in G2. There was no between-group difference (Fisherʼs exact test,* P* = 0.320, OR = 1.7, 95% CI = 0.5–5.4). All 74 dogs (G1 and G2) with abnormal joint X-ray images presented radiographic lesions bilaterally, independent of the characteristics of the lesion, with tarsal joints affected in 65 (87.8%, 95% CI = 78.4–93.4) dogs, carpal joints in 51 (68.9%, 95% CI = 57.6–78.3) dogs, elbows in 25 (33.7%, 95% CI = 24.0–45.1) dogs and stifles in 10 (13.5%, 95% CI = 7.5–23.1) dogs.

**Fig. 1:**
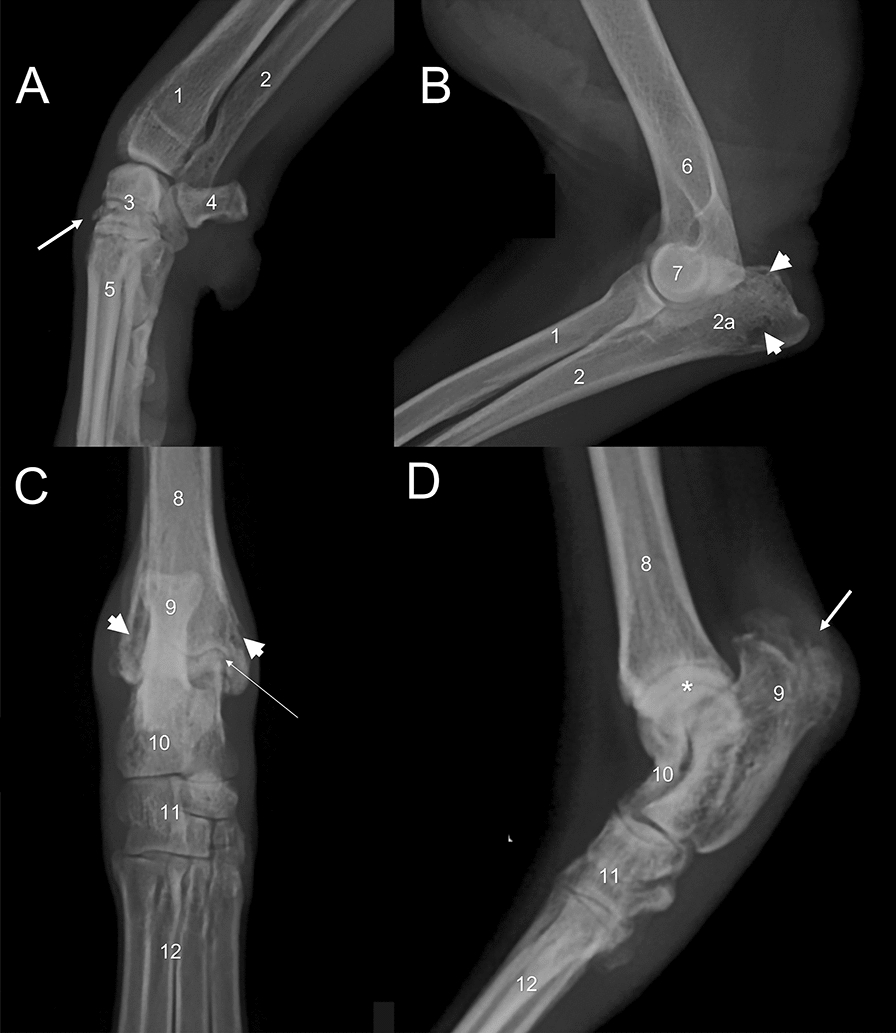
Fig. 1. Mediolateral radiography of the carpal (**A**), elbow (**B**) and tarsal (**D**) and dorsoplantar of the tarsal (**C**) joints in a dog with visceral leishmaniasis. Observe collapse of the joint spaces [arrow - (A)], bone sclerosis [arrow – (C)], evident trabecular bone [asterisk – (D)], irregular periosteal reaction [arrow – (D)], and osteolysis [arrow head – (B and C)]. 1. Radius; 2. Ulna; 2a. Olecranon; 3. Carpus bones; 4. Accessory carpus; 5. Metacarpals bones; 6. Humerus; 7. Condyle of humerus; 8. Tibia; 9. Calcaneus; 10. Talus; 11. Tarsal bones; 12. metatarsal bones.

In G1 dogs, radiographic findings observed in 162 joints with abnormalities suggestive of osteoarthritis included: (i) evident trabecular pattern in 118 (72.8%; 95% CI = 65.5–79.1) joints; (ii) subchondral bone sclerosis in 91 (56.1%; 95% CI = 48.4–63.5) joints; (iii) osteolysis in 89 (54.9%; 95% CI = 47.2–62.4) joints; (iv) soft tissue swelling around the joint (edema) in 77 (47.5%; 95% CI = 39.9–55.1) joints; (v) joint space narrowing in 58 (35.8%; 95% CI = 28.8–43.4) joints; (vi) mixed bone lesions (osteolytic–proliferative lesions) in 15 (9.2%; 95% CI = 5.6–14.7) joints; and (vi) bone proliferation in 10 (6.1%; 95% CI = 3.3–10.9) joints (Fig. 1).

In G2 dogs, radiographic findings observed in the 140 joints with abnormalities suggestive of osteoarthritis included: (i) evident trabecular pattern in 112 (80.0%; 95% CI = 72.6–85.7) joints; (ii) subchondral bone sclerosis in 73 (52.1%; 95% CI = 43.9–60.2) joints; (iii) osteolysis in 62 (44.2%; 95% CI 36.3–52.5) joints; (iv) joint space narrowing in 40 (28.5%; 95% CI = 21.7–36.5) joints; (v) soft tissue swelling around the joint (edema) in 34 (24.2%; 95% CI = 17.9–32.0) joints; (vi) mixed bone lesions (osteolytic–proliferative lesions) in 13 (9.2%; 95% CI = 5.5–15.2) joints; (vii) bone proliferation in nine (6.4%; 95% CI = 3.4–11.7) joints; and (viii) increased medullary opacity in one (0.7%; 95% CI = 00.1–03.9) joint.

Soft tissue swelling around the joint (Fisherʼs exact test, *P* < 0.0001, OR = 2.8, 95% CI = 1.7–4.6) and joint space narrowing (Fisherʼs exact test, *P* = 0.029, OR = 1.7, 95% CI = 1.0–2.7) were more commonly observed in G1 than G2 dogs. There was no between-group difference in terms of other abnormalities suggestive of osteoarthritis, such as evident trabecular pattern (Fisherʼs exact test*, P* = 0.145, OR = 0.6, 95% CI = 0.3–1.1), subchondral bone sclerosis (Fisherʼs exact test, *P* = 0.483, OR = 1.1, 95% CI = 0.7–1.8), osteolysis (Fisherʼs exact test, *P* = 0.064, OR = 1.5, 95% CI = 0.9–2.4), osteolytic–proliferative lesions (Fisherʼs exact test, *P* = 0.993, OR = 0.9, 95% CI = 0.4–2.1) or bone proliferation (Fisherʼs exact test*, P* = 0.927, OR = 0.9, 95% CI = 0.3–2.4).

### SF examination

The SF was collected without complications (sufficient volume and absence of blood in the sample) from 315 of 368 (85.5%) joints from G1 dogs and from 307 of 344 (89.2%) joints from G2 dogs. SF collected from 174 of 315 (55.2%; 95% CI = 49.7–60.6) joints from G1 dogs and from 152 of 307 (49.5%; 95% CI = 43.9–55.0) joints from G2 dogs presented an inflammatory infiltrate without a significant association between the presence of inflammatory infiltrate and the groups (neutrophils: Mann–Whitney U-test, *U*_(18)_ = 12,969, *Z* = 1.71, *P* = 0.083; mononuclear cells: Mann–Whitney U-test, *U*_(18)_ = 12,909, *Z* = − 1.78, *P* = 0.072). Inflammatory infiltrate consisted of non-degenerated and degenerated neutrophils and mononuclear cells (small lymphocytes, monocytes and macrophages), with rare observations of erythrocytes in some samples. There was no statistical between-group difference in any of the evaluated joints, in the percentage of neutrophils (tarsal: Mann–Whitney U-test, *U*_(18)_ = 880, *Z* = 1.57, *P* = 0.112; carpal: Mann–Whitney U-test, *U*_(18)_ = 921, *Z* = 0.12, *P* = 0.899; elbows: Mann–Whitney U-test, *U*_(18)_ = 686, *Z* = 1.20, *P* = 0.223; stifles: Mann–Whitney U-test, *U*_(18)_ = 757, *Z* = 0.49, *P* = 0.194) or mononuclear cells (tarsal: Mann–Whitney U-test, *U*_(18)_ = 880, *Z* = − 1.57, *P* = 0.112; carpal: Mann–Whitney U-test, *U*_(18)_ = 921, *Z* =−0.12, *P* = 0.899; elbows: Mann–Whitney U-test, *U*_(18)_ = 674.5, *Z* = − 1.31, *P* = 0.184; stifles: Mann–Whitney U-test, *U*_(18)_ = 757, *Z* = − 0.49, *P* = 0.613). The percentage of neutrophils varied from 15% to 100% (mean: 45.89 ± 22.02) in G1 dogs and from 15% to 90% (mean: 41.7 ± 20.8) in G2 dogs (Mann–Whitney U-test, *U*_(18)_ = 12,969, *Z* = 1.71, *P* = 0.083). The percentage of mononuclear cells varied from 0 to 85% in G1 dogs (mean: 53.9 ± 22.0) and from 10% to 85% (mean: 58.2 ± 20.8) in G2 dogs (Mann–Whitney U-test, *U*_(18)_ = 12,909, *Z* = − 1.78, *P* = 0.072).

**Fig. 2:**
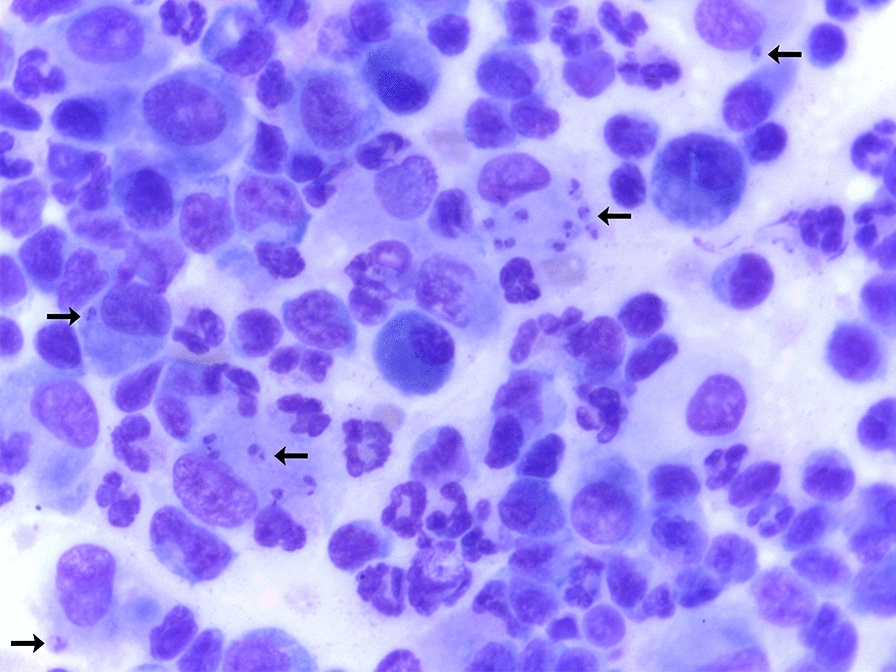
Photomicrography of a synovial fluid smear from a dog with visceral leishmaniasis showing *Leishmania* spp. amastigotes (arrows) inside macrophages (×100). Image shows mixed inflammatory infiltrate of mononuclear cells (mainly macrophages and lymphocytes) and polymorphonuclear cells (mainly segmented neutrophils)

All joints that presented SF with an inflammatory infiltrate also had radiographic evidence of osteoarthritis. Many macrophages contained magenta-staining organisms, 2–3 µm in length with a kinetoplast, compatible with *Leishmania* spp. amastigotes, were also visualized (Fig. 2). *Leishmania* spp. amastigotes were observed free and within macrophages.

**Fig. 3:**
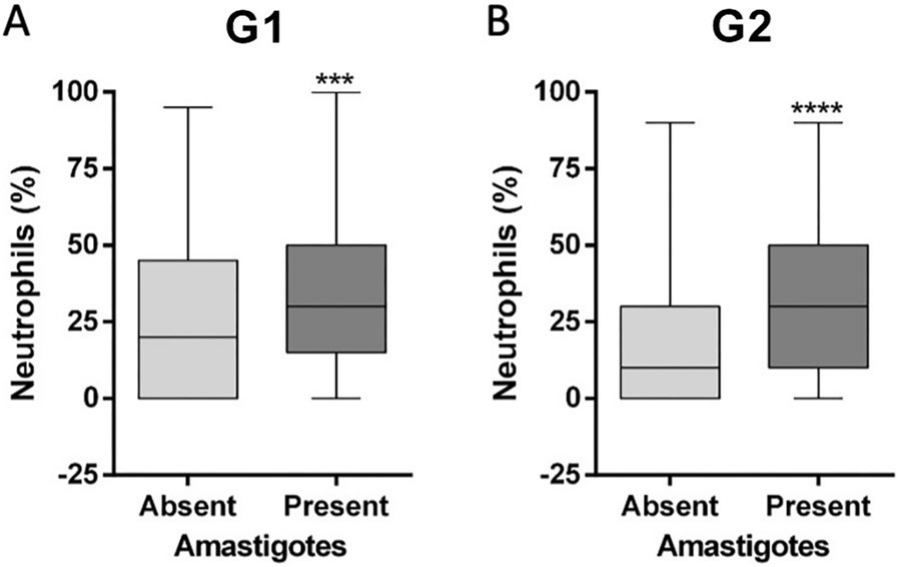
Boxplots showing the percentage of neutrophils on cytological evaluation of the synovial fluid of dogs from G1 (**a**) and G2 (**b**) dogs according to the presence or absence of amastigote forms of *Leishmania* spp. in the synovial fluid. Bars indicate minimum and maximum values, and boxes represent the first and third quartiles. Asterisks above bar indicate a statistically significant difference at ***P < 0.001 or ****P < 0.0001. G1, Group 1 dogs (CanVL and seronegative to *Ehrlichia* spp.); G2, Group 2 dogs (CanVL and seropositive to *Ehrlichia* spp.)

*Leishmania* spp. amastigotes were found in joints from 29 of the 46 (63.0%; 95% CI = 48.6–75.4) G1 dogs and from 21 of the 43 (48.8%, 95% CI = 34.6–63.2) G2 dogs, with no statistically significant between-group difference (Fisherʼs exact test, *P* = 0.177, OR = 1.7, 95% CI = 0.7–4.1); this corresponds to 169 of the 622 (27.1%; 95% CI = 23.8–30.8) joints evaluated. Amastigotes were observed in 69 of 315 (21.9%; 95% CI = 17.6–26.8) joints from G1 dogs and in 100 of 307 (32.5%; 95% CI = 27.5–38.0) joints from G2 dogs (Fisherʼs exact test, *P* = 0.002, OR = 0.5, 95% CI = 0.4–0.8) (Table 1). Per dog, the range of joints infected with *Leishmania* spp. amastigotes varied from one to eight in both groups, and the percentage of infected joints among the evaluated joints was similar (tarsal: 28.6%; carpal: 28.0%; elbow: 26.8%; stifle: 24.8%). The neutrophilic infiltrate was significantly higher in joints with *Leishmania* spp. amastigote forms in both G1 (Mann–Whitney U-test, *U*_(18)_ = 8174, *Z* = -3.76, *P* = 0.0001) and G2 dogs (Mann–Whitney U-test, *U*_(18)_ = 6543, *Z* = − 5.06, *P* < 0.0001) (Fig. 3).

**Table 1:** Absolute and relative number of joints with *Leishmania* amastigote forms in the synovial fluid of dogs from Group 1 and Group 2

Joint	Group 1	G2	Statistics
Tarsal	20/80 (25.0%)	23/70 (32.8%)	Fisherʼs exact test, *P* = 0.288, OR = 0.6, 95% CI = 0.5–1.1
Carpal	16/77 (20.7%)	28/80 (35.0%)	Fisherʼs exact test, *P* = 0.047*, OR = 0.4, 95% CI = 0.2–0.9
Elbow	18/82 (21.9%)	25/78 (32.0%)	Fisherʼs exact test, *P* = 0.149, OR = 0.5, 95% CI = 0.2–1.2
Stifle	15/78 (19.2%)	24/79 (30.3%)	Fisherʼs exact test, *P* = 0.239, OR = 0.6, 95% CI = 0.3–1.3
Total	69/315 (21.9%)	100/307 (32.5%)	Fisherʼs exact test, *P* = 0.002*, OR = 0.5, 95% CI = 0.4–0.8

* CI* Confidence interval,* OR* odds ratio

*Statistically significant

^a^ Group 1 (G1) dogs had canine visceral leishmaniasis (CanVL) and were seronegative to *Ehrlichia* spp.; Group 2 (G2) dogs had CanVL and tested seropositive to *Ehrlichia* spp.

^b^Values are presented as the number of samples with *Leishmania* amastigotes/number of samples collected, with the percentage given in parentheses

## Discussion

Immune-mediated polyarthritis (IMPA) is the most common form of inflammatory joint disease found in dogs. Differential diagnosis of secondary IMPA includes vector-borne diseases (specifically those caused by *B. burgdorferi* and *L. infantum*) and sulfonamide-containing medications [19]. Additionally, *E. canis*-related polyarthritis has been rarely reported in dogs [14, 15], although no previous studies have consistently supported the occurrence of this clinical sign in CME [16, 17]. In Brazil, *E. canis* is the most common and widespread tick-borne pathogen in dogs [20]. Although cases of borreliosis-like disease have been reported in humans and animals by western blotting and enzyme immunoassay tests [21], to date *Borrelia* spp. has never been detected by molecular methods or isolated by cell culture in dogs in Brazil, and there is no conclusive evidence on the potential tick species associated with the transmission of those *Borrelia*-like organisms.

The authors have not observed dogs with *E. canis*-related polyarthritis in their clinical routines and veterinary practices. Dogs from the present study were divided into two groups according to the presence or absence of anti-*Ehrlichia* spp. antibodies, since the suppression of the immune system in dogs with clinical leishmaniasis could enable the reactivation of a previously subclinical *E. canis* infection [3], and there can be a synergism between *L. infantum* and *E. canis* during co-infection in dogs that can worsen the dog's clinical condition [3]. Although the presence of anti-*Ehrlichia* antibodies does not confirm an active infection and might merely be indicative of previous exposure to *Ehrlichia* spp., in the present study it was not possible to perform a molecular diagnosis to confirm *Ehrlichia* infection status due to financial constraints.

In the present study, the prevalence of CanVL-associated arthritis in dogs was 83.1%, with no difference between groups regarding the percentage of dogs with joint abnormalities on radiography. This prevalence is similar to 91.3% observed in a study conducted with 46 dogs with clinical leishmaniasis independent of the presence of clinical signs of arthritis [13]. Nevertheless, it is much higher than 4.6% [6] and 44.8% [4] observed in two previous studies with dogs with polyarthritis, possibly because radiographic evaluation and SF analysis were performed in eight joints independently of clinical signs of arthritis.

Although some studies have described cases of monoarthritis associated with CanVL [6, 9, 12, 22, 23], in the present study all dogs presented involvement of multiple joints, as has been observed in other studies [4, 7–9, 13, 23]. All evaluated joints were affected bilaterally, but the radiographic changes and the intensity of the inflammatory infiltrate were not the same on both sides. Bone lesions have probably a hematogenous origin, which may explain the presence of bilateral joint involvement [4].

The most affected joints were tarsal (87.8%), followed by carpal (68.9%), elbows (33.7%) and stifles (13.5%), as observed in a previous study in which the same joints were evaluated and the inclusion criterion was the diagnosis of CanVL regardless of clinical signs of arthritis [13]. In other studies in which the inclusion criterion was the presence of clinical signs of arthritis, the joints most frequently affected were the carpal [6] and the carpal and stifles [4].

Radiographic patterns of articular lesions were similar between groups evaluated in present study, with most of the dogs from both groups presenting erosive lesions; these results confirm previous findings [4, 6, 8, 10, 11]. Proliferative and mixed (osteolytic and proliferative) lesions were also visualized in both groups, without any evident between-group difference, as also has been reported by other authors [4, 7, 9–11, 22–24]. Dogs from G1 were more likely to present soft tissue swelling around the joint (*P* < 0.0001) and joint space narrowing (*P* = 0.029); however, we hypothesized that this was just an incidental finding. It has been proposed that subacute or acute stages are associated with non-erosive and chronic stages related to erosive lesions [4].

Although the evaluation of SF in dogs with polyarthritis secondary to *Leishmania* infection has been previously reported [6, 7, 9, 11, 25–27], to the best of our knowledge this is the first study to perform SF evaluation in a large number of dogs (*n* = 89) with CanVL, with and without previous exposure to *Ehrlichia* spp., independent of the presence of clinical signs of arthritis. Overall, previous studies have reported an inflammatory infiltrate on SF smears, with either a preponderance of neutrophils [6, 9] or mononuclear cells [4, 7, 11], with *Leishmania* spp. amastigotes observed on either neutrophils, macrophages or free on the SF [6, 7, 9, 11, 25–27]. We found no significant differences in the type of inflammatory infiltrate on SF between joints or between groups. However, there was a slight predominance of mononuclear cells over neutrophils in the inflammatory infiltrates in both groups, as previously reported in a study evaluating SF of dogs with VL [4]. *Leishmania* spp. amastigotes were found in 27.1% of the joints evaluated, more frequently in joints from G2 dogs (*P* = 0.002). Although the authors can only assume that G2 dogs had been previously exposed to *Ehrlichia* spp., it is well known that co-infection between *L. infantum* and *E. canis* is common and may alter clinical manifestations [3]. Further studies evaluating SF in dogs co-infected with *L. infantum* and *E. canis* should be performed to evaluate this finding.

## Conclusions

In conclusion, the present study found a high prevalence of arthritis in dogs with CanVL, with the involvement of multiple joints in all dogs. The most affected joints were tarsal, followed by carpal, elbows, and stifles. There was a slight predominance of mononuclear cells over neutrophils in the inflammatory infiltrates on SF in both groups. Although no difference was observed between groups related to the number of dogs with polyarthritis, most of the radiographic findings and the presence of an inflammatory infiltrate in SF, *Leishmania* spp. amastigotes were found more frequently in joints from G2 dogs. Further studies evaluating SF in dogs co-infected with *L. infantum* and *E. canis* should be performed to evaluate this finding.

## Data Availability

The datasets used and/or analyzed during the current study are available from the corresponding author upon reasonable request.

## Abbreviations

CanL: Canine visceral leishmaniasis
CME: Canine monocytic ehrlichiosis
ELISA: Enzyme-linked immunosorbent assay
G1: Group 1
G2: Group 2
IMPA: Immune-mediated polyarthritis
SF: Synovial fluid
